# *Endocrine Oncology*: the home for translational research in cancer and hormones

**DOI:** 10.1530/EO-23-0012

**Published:** 2023-05-12

**Authors:** Justo P Castaño

**Affiliations:** 1 Maimonides Biomedical Research Institute of Córdoba (IMIBIC), Córdoba, Spain; 2 Department of Cell Biology, Physiology, and Immunology, University of Córdoba, Cordoba, Spain; 3 Reina Sofía University Hospital, Cordoba, Spain; 4 Centro de Investigación Biomédica en Red de Fisiopatología de la Obesidad y Nutrición, (CIBERobn), Cordoba, Spain

The intense activity within biomedical research has never been so populated and voluminous worldwide. As a consequence, publishing research articles in respected journals in the biomedical field has become a challenging endeavour. As scientists of our time, the search for suitable visibility is a primary goal, not an aesthetic or superfluous option. We are required by the research community, the funding bodies, and society at large, to find the right niche to reach our optimal target audience of colleagues and peers; those with whom to share, discuss and leverage the results from our research and, ultimately, bring it closer to the patients. In this context, I sincerely believe that the founding of *Endocrine Oncology* in February 2021 was a timely and sound decision. The journal has adequately progressed over these two years, completing the initial technical requirements despite a demanding environment, from the world health threat of the COVID-19 pandemic to the changes and challenges in the editorial and publishing arena. With *Endocrine Oncology’s* recent acceptance to PubMed, we now face an exciting period of journal development and consolidation. This is a critical time to define the personality of a journal and transform it into a useful resource for the research community.

*Endocrine Oncology* was born as a sister journal of *Endocrine-Related Cancer,* and is evolving into a valuable new target for an active community of translational researchers working on distinct disciplines that converge at the interface of endocrine tumours and hormone-related cancers. Opportunities for novel discoveries and their translational application often blossom at the interface of fields. *Endocrine Oncology* is a necessity forged by the pragmatic daily interaction between cooperating disciplines managing and studying these tumours, which are not limited to the obvious Endocrinology and Oncology, but involve a plethora of experts, from Cellular and Molecular Biologists, and Biochemists to Pathologists, Radiologists, Specialists in Nuclear Medicine or Internal Medicine, Surgeons, Nurses, Urologists, Geneticists, Nutritionists, Bioinformaticians, Gastroenterologists and so on. This ample array of experts reflects the complexity and diversity of hormone-related tumours, which break the boundaries of established fields and require a multifaceted, prismatic, and integrative view. This is the spirit that explains the birth and growth of *Endocrine Oncology*.

The mission of a journal is to help the research community to convey its scientific results and conceptual advances to our peers and society in a rigourous manner that stands the test of time. *Endocrine Oncology* is committed to assist authors to achieve this goal: bring their best discoveries to an audience avid for novel information through a balanced combination of efficiency, rigour, transparency and timeliness. The long-term success of a journal relies on the persisting soundness and reliability of its published studies. Our overarching goal is that *Endocrine Oncology* becomes an appreciated asset for translational researchers working at the interface of hormones and cancer, where their published work will stand the test of scientific time.

To achieve such high expectations, *Endocrine Oncology* relies on four sound pillars. Firstly, the authors in this emerging field, a community that gains a specific voice for its results. Secondly, an experienced Editorial Board assisted by expert reviewers, comprising a comprehensive collection of different disciplines and involving a balanced representation of geographical distribution. Thirdly, the professional and enthusiastic support of a publisher, Bioscientifica, which cares not just for economic profit but for the major goal of a well-established Society for Endocrinology, i.e., to ensure the progress of sound scientific research. Finally, our audience, the readership, those who are the target and owners of the original, and relevant scientific knowledge that we aim to publish at *Endocrine Oncology*.

Innovation is the fuel that fosters advancement of research, and so is a capital resource to develop a new journal. Endocrine-related tumours and cancers have often lagged behind other more prevalent diseases in terms of attention and funding. Consequently, the application of novel technical and scientific approaches has been slower in this field, delaying the pace of discovery. *Endocrine Oncology* aims at and encourages the incorporation of these advances into translational research in hormones and cancer: from single-cell technologies and spatial transcriptomics, to novel clinical applications of technologies involving mRNA, CRISPR, base editing, immunotherapies, nanotechnologies, biocomputational studies, artificial intelligence and deep learning.

*Endocrine Oncology* will continue to consider for publication all articles that contribute to the advancement of the field and are scientifically sound. All aspects of the field will be considered, from neuroendocrine tumour to thyroid and the interplay of diabetes, obesity, metabolism and cancer. *Endocrine Oncology* encourages studies focusing on methodological and technical advances including bio-computational approaches, genomics, proteomics and transcriptomics. Clinical studies and proof of concepts are also welcome, along with translational studies in the development of novel therapies.

The necessarily transdisciplinary nature of *Endocrine Oncology* is both a strength and an opportunity. It provides a unique profile to receive the best science in this largely cooperative field, while uniting in a single home studies that are often dispersed in journals of distant disciplines that can remain out of sight. The key to making our journal useful and valuable will be to integrate in a balanced and harmonised manner those different fields and studies, while attracting and maintaining studies with high levels of originality, quality and relevance.

The thrilling present and promising future for *Endocrine Oncology* research lies ahead, beginning with the appointment of a new Deputy Editor, Prof Simona Glasberg, MD, Director of the ENETs Centre of Excellence, the Hebrew University of Jerusalem, Israel. With her comprehensive experience, Prof Glasberg will focus on developing the clinical aspect of the journal, ensuring we continue to publish work at the forefront of endocrine-related oncology. Alongside *Endocrine Oncology’s* recent acceptance to PubMed and the Directory of Open Access Journals, the journal moves into its next exciting stage of development. Applications for Web of Science and Scopus are both currently in review.

As we work to create a high-impact journal fit not only for the present but the future endocrine and oncology communities, we invite you to consider *Endocrine Oncology* as the home for your research.

Justo P Castaño

Editor-in-Chief

